# Examining Job Complexity on Job Crafting Within Conservation of Resources Theory: A Dual-Path Mediation Model

**DOI:** 10.3389/fpsyg.2021.737108

**Published:** 2021-10-07

**Authors:** Jing Yi Bai, Qing Tian, Xia Liu

**Affiliations:** ^1^School of Business, Macau Univerity of Science and Techology, Macau, Macao, SAR China; ^2^School of Business, Macau University of Science and Technology, Taipa, Macao, SAR China; ^3^School of Humanities and Management, Southwest Medical University, Luzhou, China

**Keywords:** job complexity, work engagement, energy depletion, approach crafting, avoidance crafting

## Abstract

This study examined the different ways in which job complexity influences employees’ job crafting. Specifically, we draw on conservation of resources (COR) theory to hypothesize that job complexity is positively related to approach crafting *via* work engagement (i.e., resource gain process). At the same time, job complexity may also induce employees to engage in avoidance crafting (i.e., resource loss process) as employee energy resources are depleted. Our data consist of 251 employees working in Macau. We used structural equation modeling (SEM) in Mplus software to test the proposed hypotheses. Our findings confirm that job complexity has differential effects on approach and avoidance crafting through work engagement and energy depletion. These findings highlight the importance of costs and benefits of job complexity and the importance of resources in the employees’ job crafting process. We discuss the practical implications for modern organizations in which complex jobs are prevalent.

## Introduction

The radical changes in the work environment deriving from the COVID-19 pandemic have been challenging the traditional work design (Wang et al., 2021). Today, job holders often face increasingly complex job demands resulting from the increasing fluidity in employment arrangements (Ingusci et al., 2021). Job complexity refers to jobs that are mentally challenging and therefore require the use of an employee’s personal resources to cope with the amount of stress involved (Sacramento et al., 2013; Sung et al., 2017). Such demanding jobs often induce employees to work faster and longer or to alter their work habits (Ragu-Nathan et al., 2008). Therefore, highly complex jobs push employees to develop new strategies to craft their jobs so that they can acquire and conserve their valuable resources. Employees’ proactivity to adapt to job requirements and craft their jobs is thus becoming more important than ever (Wrzesniewski and Dutton, 2001; Tims and Bakker, 2010; Zhang et al., 2021).

Job crafting can be either approach- or avoidance-oriented (Bruning and Campion, 2018; Bindl et al., 2019; Zhang and Parker, 2019; Costantini et al., 2021). More specifically, in approach crafting employees accept the challenge stressors, increase resources, or improve work experience, whereas in avoidance crafting employees seek to withdraw from the job work roles (Bruning and Campion, 2018). Existing studies found that employees tend to favor the approach crafting (Lichtenthaler and Fischbach, 2019, p. 31; see also the empirical study Cenciotti et al., 2017; Petrou et al., 2017). Our study considers both approach and avoidance crafting (Bruning and Campion, 2018) because employees’ approach and avoidance crafting can be triggered by certain psychological states simultaneously (Lichtenthaler and Fischbach, 2019). Moreover, despite existing research on the impacts of job characteristics (such as task complexity) on job crafting (e.g., Ghitulescu, 2007), the mechanisms underlying the relationship between job complexity and employees’ approach and avoidance crafting are unclear. Our research examines whether and how job complexity affects employees’ job crafting through a dual-mediation pathway.

We draw upon conservation of resources (COR) theory to investigate how job complexity leads to distinct forms of job crafting, resulting in approach and avoidance strategies. COR theory assumes that individuals’ resources are salient factors in explaining individuals’ coping responses when confronted with demanding situations (Hobfoll, 1989). When dealing with complex jobs and potential threats to their resources and wellbeing, employees will actively strive to acquire additional resources or conserve resources (Hobfoll, 1989; Harju et al., 2016). According to COR theory, complex jobs may enhance individuals’ intrinsic interest in finding meaning in their work (Cavanaugh et al., 2000; Chung-Yan, 2010), which can further energize them to grow and achieve. Job complexity represents a strong motivational force in the work setting (Shalley et al., 2009; Sung et al., 2017), leading to employees’ resource gain. Employees feel especially engaged in their work when personal growth and achievements meet with increased efforts dealing with complicated tasks (Breevaart and Bakker, 2018). We examine the role played by work engagement to increase resource gain and its importance for the relationship between job complexity and job crafting. Work engagement is a positive motivational state that combines high energy with a strong intention to invest one’s resources to work (Schaufeli and Bakker, 2010). This engaged state enables employees to cope with complex jobs more proactively and causes them to craft their jobs employing the approach strategy way. Employees that are highly engaged are energetic and enthusiastic about their work and, therefore, may be more welcoming to challenging work activities (Rasool et al., 2020).

Nevertheless, complex jobs are challenging (Sung et al., 2017; Pan and Sun, 2018). As the increasing complexity of tasks increases exploitation, employees may not cope with increasing demands due to limited personal resources (Xie and Johns, 1995; Hakanen et al., 2006). Complex jobs bring high expectations and responsibilities to employees and require them to invest extra time and effort to cope with the assignments. This burdensome process drains employees’ valuable personal resources, resulting in resource loss (Pan and Sun, 2018) as employees are trying to cope with the demanding and potentially challenging assignments (Halbesleben et al., 2014). When employees have insufficient resources to cope with increasingly demanding tasks, they eventually feel very overextended and withdraw from their work role, i.e., reduce, or eliminate part of their works to protect and retain their resources (Nielsen, 2013; Harju et al., 2016). Based on the resource gain and resource loss processes of COR theory, we examine how work engagement and energy depletion affect the relationship between job complexity and job crafting.

This research contributes to job crafting theory by exploring the antecedents of employees’ approach and avoidance crafting, responding to the calls by Zhang and Parker (2019) for examining the “variables that predict all types of job crafting (including approach and avoidance types) in the same direction (p. 140).” Our study considers both the approach and avoidance strategies to job crafting and synthesizes their antecedents to fill this knowledge gap. By examining the integrated and comprehensive framework of job crafting, our study provides a more nuanced and systematic view of the job crafting phenomenon by examining the underlying mechanisms that govern the relationship between job complexity and approach/avoidance crafting.

Furthermore, our study offers a balanced view to exploring the dual effects of job complexity. We propose that complex jobs may – on the one hand – enhance work engagement, which in turn increases approach crafting through resource gain and – on the other hand – lead to energy depletion, which increases avoidance crafting through resource loss. Thus, our study provides a better understanding of the motivational and strain mechanisms related to job complexity. By exploring these mechanisms, we challenge existing assumptions according to which job complexity primarily energizes employees in the workplace (for reviews, see Ilgen and Hollenbeck, 1991; Morgeson and Campion, 2003). At the same time, we propose that job complexity depletes employees’ psychological energy resources. Managers should be aware of the trade-offs of job complexity that may represent threats to employee wellbeing (Karasek, 1979). This may require a change in managers’ mindsets which may in turn influence job design in an era when challenging jobs are becoming increasing prevalent.

Lastly, by exploring the dual processes of COR theory, this study takes a closer look at job crafting from the resource gain and resource loss perspective and makes novel predictions regarding approach and avoidance crafting. Work engagement represents a resource gain process in which employees actively deal with complex jobs, whereas energy depletion represents a resource loss process in which employees reduce and avoid stressful job demands. We suggest that job complexity not only motivates approach crafting *via* increased work engagement but also triggers avoidance crafting *via* increased energy depletion.

This paper is organized as follows. First, we discuss the resource gain and resource loss processes of COR theory and how approach and avoidance crafting act as a behavioral manifestation of individuals current resources. Then, we develop our main hypotheses and the dual-path mediation model to test our hypotheses before presenting a detailed account of the methodology and results. Finally, we discuss the implications of our results and point out possible limitations and future research directions.

### Theoretical Background and Hypothesis Development

#### Theoretical Background

Conservation of resources theory assumes that individuals are highly motivated to accumulate additional resources for the future. COR theory contains the concepts of “resource gain” and “resource loss” processes (Hobfoll, 1989) which present two predictions regarding the use of approach and avoidance job crafting strategies in the workplace. “Resource gain” suggests that individuals employ the approach strategy to acquire additional structure and social resources (e.g., gaining opportunity to develop oneself and asking for feedback; Tims et al., 2012). “Resource loss” occurs when individuals with highly complex and demanding jobs use avoidance crafting to protect and conserve their resources. Challenging tasks may require additional time and energy which can lead to employees engaging in avoidance crafting to reduce the loss of valuable resources. Thus, based on COR theory, employees may adopt approach and avoidance crafting selectively and strategically to deal with complex jobs.

There are two job crafting conceptualizations, namely, resource-based and role-based perspectives, which differ in job crafting types and job crafting motives (Wrzesniewski and Dutton, 2001; Tims and Bakker, 2010). Researchers have generally focused on the approach aspect of job crafting (e.g., expansions of task boundaries). Little is known about the motives that lead employees to apply an avoidance strategy, i.e., to reduce their work role boundaries. Recent studies that have integrated both crafting frameworks tend to agree that employees both expand (i.e., approach crafting) and reduce (i.e., avoidance crafting) their job boundaries (e.g., Bruning and Campion, 2018; Lichtenthaler and Fischbach, 2019; Zhang and Parker, 2019). Approach crafting activities are active, effortful, motivated, and directed toward positive aspects of work. In contrast, avoidance crafting involves avoiding or escaping from negative aspects of work. However, there is no empirical evidence regarding the mechanisms behind the relationship of complex jobs and approach and avoidance crafting. Individuals will acquire new resources to achieve goals and engage in activities to acquire additional resources (i.e., resource gain; Hobfoll, 1989; Halbesleben et al., 2014). Approach crafting is instrumental in acquiring extra resources to seek positive aspects of work (i.e., work role expansion, social expansion, work organization, adoption, and metacognition; Bruning and Campion, 2018). Specifically, work role expansion refers to how employees can extend their work role beyond their formal job description. Social expansion involves the proactive use of social resources involving their colleagues and supervisors. Work organization refers to employees proactively designing and organizing their work and surroundings. Employees can also craft their jobs through adoption, i.e., using technology and knowledge to enhance the work process. Metacognition captures employees’ cognitive sensemaking about their jobs and represents the manipulation of their psychological state. Employees performing highly complicated jobs may be most conducive to increased approach crafting behavior to gain additional resources (Kuijpers et al., 2020). On the other hand, individuals protect the limited personal resources and prevent them from becoming depleted (i.e., resource loss) through avoidance crafting (i.e., withdrawal crafting and work role reduction). Withdrawal crafting suggests that employees may remove themselves from a person, situation, or event either mentally or physically, while employees may also consciously and proactively reduce their work role responsibility (i.e., work role reduction; Bruning and Campion, 2018). Employees with complex jobs may engage in avoidance crafting to protect existing resources (Hobfoll, 2001; Halbesleben and Bowler, 2007). Thus, our theoretical model suggests that job complexity leads to both resource gain and resource loss processes, which may, in turn, lead to approach and avoidance crafting.

### Job Complexity and Approach/Avoidance Crafting

Conservation of resources theory (Hobfoll, 1989) assumes that individuals acquire additional resources to meet challenging tasks when they perceive potential personal growth and resource gains. Resources aid the process of growth and gain, because initial resource promotes future gain, thus generating “gain spirals” (Chen et al., 2015 p. 97). Based on COR theory, we argue that job complexity is positively related to approach crafting. Job complexity facilitates thinking skills and triggers employees’ initiatives to acquire more knowledge, information, and support from colleagues (Sung et al., 2017). Thus, complex jobs will most likely help employees develop their skills and gain additional resources from their work environment. In this view, job complexity motivates and legitimates employees’ self-job redesign and self-management, such as job crafting (Hornung et al., 2010; Ohly and Fritz, 2010; Petrou et al., 2012). Complex jobs also increase task motivation and enable employees to exhibit skills and novel approaches to solve problems. Employees will be prompted to craft their job to fulfill the required tasks (Frese et al., 2007; Tims et al., 2013; Kuijpers et al., 2020).

Tims and Bakker (2010) suggested that complicated tasks will increase employees’ ability to identify alternative opportunities and strategies to obtain additional resources. Under challenging job conditions, employees focus on acquiring resources and on investing their resources to gain additional resources through job crafting (Nielsen, 2013; Harju et al., 2016). For example, employees need to process information and experience new problems, which often create constructive interaction with supervisors or other colleagues that provide help, support, and guidance. Thus, approach crafting aiming at acquiring resources may represent a favorable way to handle complex jobs.

However, some employees with complex jobs may employ an avoidance strategy. According to the resource loss process in COR theory, employees exposed to a stressful situation tend to have a more negative work attitude (Samma et al., 2020). Job complexity prompts employees to experience strain and use avoidance crafting to reduce their strains. Complex jobs create extensive responsibilities and strain employees’ time and efforts, depleting their energy and resources (Ito and Brotheridge, 2003). Employees may want to reduce demands to reduce resource loss. Therefore, employees with highly complex jobs may employ escape/avoidance strategies to ease their strains.

Building on COR theory, we posit that approach crafting represents a resource gain process as employees deal with challenging job demands that motivate them, whereas avoidance crafting constitutes a resource loss process when employees feel that such job demands are overly stressful. Employees are inclined to choose tasks at which they will perform well and that are not too difficult (Elliot and Thrash, 2001). Approach crafting seeks to obtain and retain the positive aspects of work and gives employees an opportunity to demonstrate their competence (Elliott and Dweck, 1988; Harju et al., 2021). However, avoidance crafting occurs when employees try to avoid potential failure when handling certain tasks which may have them appear incompetent (Harju et al., 2021). Thus, employees may avoid certain aspects of complex jobs while approaching others. We suggest the following hypothesis:

*Hypothesis 1*: Job complexity is positively related to approach crafting.

*Hypothesis 2*: Job complexity is positively related to avoidance crafting.

### Job Complexity and Work Engagement

Unlike routinized jobs, complex jobs provide employees with more opportunities to explore, exercise control, and be responsible for outcomes (Pierce et al., 2009), generating positive states of vigor, dedication, and absorption (i.e., work engagement). Job complexity satisfies individuals’ desire to learn and achieve at work since complex jobs offer the kinds of opportunities and internal rewards they value. Specifically, complex jobs are more malleable and accessible in making changes and employees are encouraged to consider alternative solutions to handle them (Pierce et al., 2009). Thus, employees are more likely to invest more of themselves (e.g., their resources, time, and efforts) into the job (Brown et al., 2014). Moreover, complex jobs are stimulating and challenging, which may trigger employees to invest additional efforts to fulfill achievements and lead to the motivational process (Chung-Yan, 2010). Such resource gain process triggered by job complexity results in the positive effects that include personal growth and development (LePine et al., 2005). Consequently, those employees may experience a high level of job meaningfulness (Bunderson and Thompson, 2009), which in turn will be reflected in a heightened sense of dedication.

Further, job complexity can enhance employees’ absorption in their jobs because challenging jobs require a high level of information-processing capacity (Gardner and Cummings, 1988; Bledow et al., 2011). Complex jobs require individuals to become both self-absorbed and self-revealing to allocate work-related resources actively toward the tasks (Rich et al., 2010). In contrast, simple or routine jobs may cause boredom, distract employees from their tasks, and reduce their interest in tasks (Fisherl, 1993), resulting in a lower level of work engagement (Gorgievski and Hobfoll, 2008). As such, job complexity triggers resource gains and fosters work engagement.

*Hypothesis 3*: Job complexity is positively related to work engagement.

### The Mediating Role of Work Engagement

More specifically, we hypothesize that work engagement plays a mediating role between job complexity and approach crafting behavior. According to COR theory, employees are motivated to accumulate and obtain valuable resources to deal with challenging job demands (i.e., resource gain process of COR theory). Complex jobs allow employees to personalize their tasks because confronting tough tasks enables them to seek new solutions to complete their jobs (Brown et al., 2014). Therefore, individuals experiencing complex jobs might craft their jobs more proactively because they can acquire additional resources to deal with challenging job demands through approach crafting. As dedicated employees are more likely to exert additional efforts to seek resources and information, employees with positive psychological functioning (i.e., high work engagement) are able to enhance their job crafting (Bakker and Demerouti, 2017). Approach crafting enables employees to accumulate additional tangible and intangible resources to reduce uncertainty and gain a strong social support network (Robinson and Griffiths, 2005); this enhances their productivity and enables them to cope with demanding and complex jobs.

Further, employees fully immersed in their work can use their personal resources more efficiently and tend to be more focused on their work (Chang et al., 2013). Employees immersed in their jobs are more likely to seek out new perspectives, challenges, and solutions. Besides, their immersion motivates employees to focus on work activities and be more persistent to achieve their goals. As a result, individuals feel motivated to invest themselves in their work and acquire the resources needed to overcome potential challenges (Aubé et al., 2009); all these endeavors facilitate approach crafting at work. Moreover, when individuals with challenging jobs are vigorous, they are more likely to consider problem-solving choices and actions to overcome their challenges (Barsade, 2002). This augmented positive cognitive state is an essential motivation for approach crafting because it allows individuals to build a new association between the job and the required resources.

Employees tend to acquire and invest resources to maximize their psychological energy resources (Halbesleben et al., 2014). Those with larger psychological resources can achieve their goals and thrive through the resource gain process. Employees with a high level of engagement will be motivated to gain additional resources through approach crafting actions, such as incorporating challenging tasks or seeking other social resources (Tims et al., 2012; Niessen et al., 2016). In line with the above discussion, we hypothesize that there exists a relationship between job complexity and approach crafting through work engagement.

*Hypothesis 4*: Work engagement mediates the positive relationship between job complexity and approach crafting.

### Job Complexity and Energy Depletion

Complex jobs do not always lead to positive psychological functioning but can lead to a negative psychological state, such as energy depletion. Employees with complex jobs may attempt to conserve energy in order to recover resource losses states (Samma et al., 2020). When employees fail to achieve a challenging goal, they will seek to conserve resources by reducing efforts for tasks that consume their resources (Hobfoll, 1989) and protect themselves from strains by reducing resource losses when employees feel hard to meet the requirements of challenging jobs and gain resources (LePine et al., 2005). Complex jobs may impose psychological and cognitive pressure on employees (Sung et al., 2017), leading to resource loss and energy depletion which most likely occurs when individuals possess inadequate resources or insufficient resource gain to meet work demands. Thus, job complexity potentially drains valuable resources, which leads them to experience physical and psychological exhaustion (Chung-Yan, 2010). Based on this reasoning, we propose that stressful situations caused by complex jobs are likely to wear out an employee’s psychological energy resources.

*Hypothesis 5*: Job complexity is positively related to energy depletion.

### The Mediating Role of Energy Depletion

Our study also proposes that job complexity results in a resources loss process, leading to energy depletion. According to COR theory’s resource loss process, individuals with complex jobs may craft their jobs less proactively to conserve resources. As workload, accumulation can cause employees feel overwhelmed and unable to deal with their tasks. As a result, they will feel frustrated and exhausted (Samma et al., 2020). By reducing their workload or withdrawing from their work role, they may then try to reduce strain and protect their valuable resources (Tims et al., 2012). Exhausted employees tend to display avoidance crafting because they cannot regulate their energy successfully (Demerouti et al., 2005) or adapt to their depleting resources (Wright and Hobfoll, 2004). Individuals who are in the condition of energy depletion might be more likely to withdraw from their job role to protect existing personal resources.

**Figure 1:**
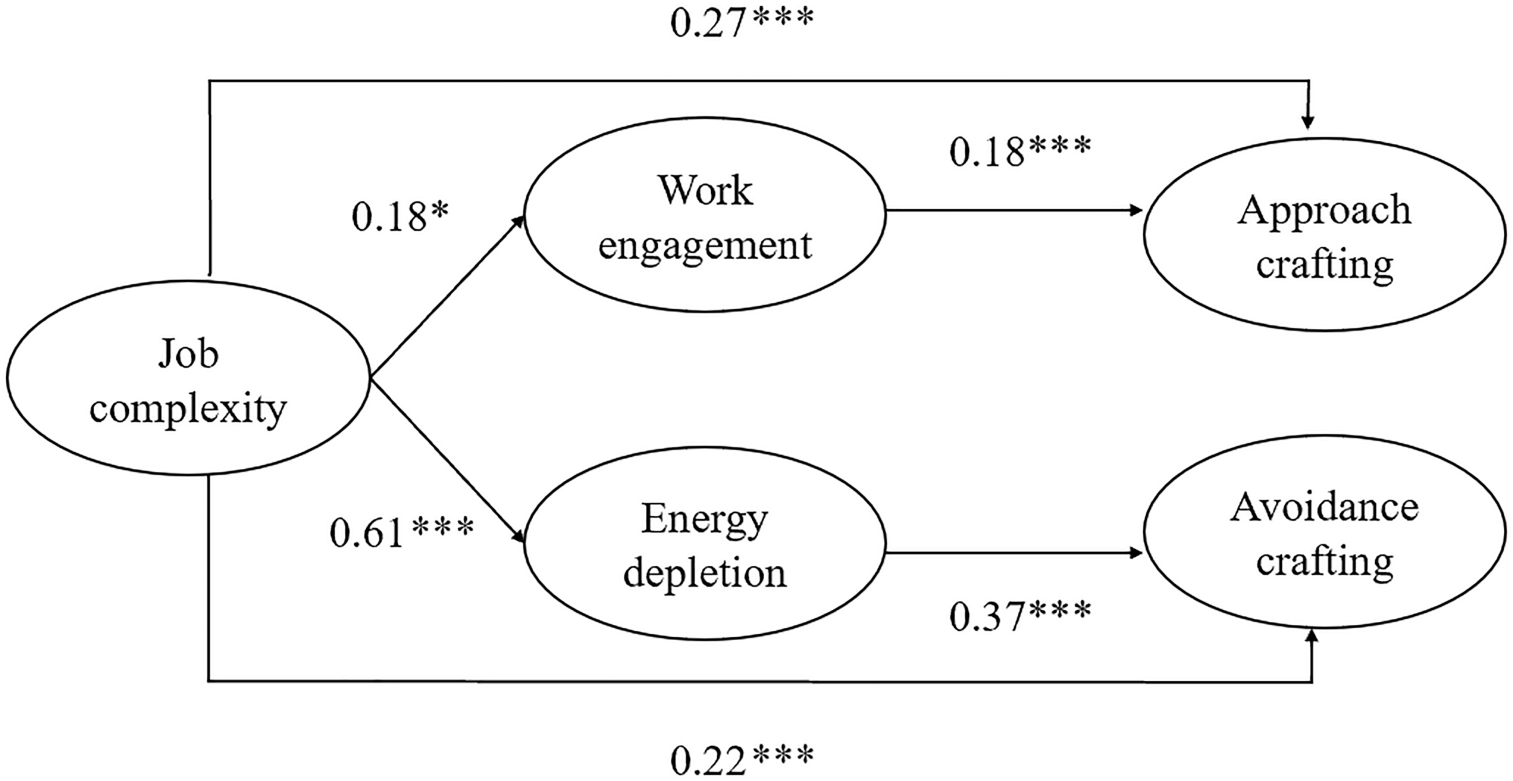
Path analysis results ^*^p<0.05 and ^***^p<0.001.

Employees with challenging jobs may experience a depletion of resources and will attempt to make their situations less overwhelming in the dysfunctional state of energy exhaustion (Hobfoll, 1989). Employees in the energetically depleted state may have difficulties recognizing opportunities and challenges but may also avoid challenges (Swider and Zimmerman, 2010). Avoidance crafting represents any efforts by employees to evade challenging job demands. It constitutes a resource loss process, where employees seek to mitigate the straining effects of work to protect their resources and improve their wellbeing (Harju et al., 2021). Since approach crafting requires additional efforts (Zhang and Parker, 2019), employees may adopt the avoidance strategy to relieve stress. The resource loss process of COR theory suggests that individuals with demanding jobs might be less likely to engage in behavior that consumes their resources (Hobfoll, 1989). We suggest the existence of an indirect relationship between job complexity and avoidance crafting through energy depletion. Figure 1 summarizes the proposed research model of this study.

*Hypothesis 6*: Energy depletion mediates the positive relationship between job complexity and avoidance crafting.

## Materials and Methods

### Participants and Procedures

We tested our hypotheses with a sample of employees from six companies covering a variety of occupations and industries in Macau. Participants were recruited by contacting the human resource managers of the participating companies, requesting their assistance in our study. Using a questionnaire-based survey (e.g., Rasool et al., 2020), we collected data at three time points to reduce common method bias (Podsakoff et al., 2003). The questionnaire included the following parts: (i) a mention of the study purpose and privacy statements, (ii) the possible answers on a 5-point Likert scale, and (iii) the respondents’ demographic information. Respondents were notified that participation in the survey was voluntary, confidential, and anonymous, as we did not use clear names but identification codes known only to the authors.

At Time 1 (T1), we distributed the paper-and-pencil survey to 400 employees and asked them to rate their job complexity level, receiving the completed survey from 367 participants (91.8% response rate). Approximately 2 weeks later after the participants had completed the Time 1 survey (at Time 2), they were asked to rate their work engagement and energy depletion. We chose to separate both questionnaires by a 2-weeks interval because our model deals with a psychological process and its effects on employee behavioral outcomes (for a similar approach see Deng et al., 2018). A total of 323 employees (88.0% response rate) returned the T2 survey. After an additional 2 weeks (at time T3), we sent out the last questionnaires to obtain information regarding the levels of the participants’ approach and avoidance crafting. From the 292 returned questionnaires (90.4% response rate), 41 had to be discarded due to missing or randomly filled data (25) and unmatched responses (16), resulting in an overall response rate of 62.8% (i.e., 251 completed data sets from 400 questionnaires that had initially been sent out.

The 251 included participants had an average age of 42.12 years (*SD*=12.37), an average organizational tenure of 6.95 years (*SD*=6.15), and 51% of them were females. Their highest education levels were high school diploma (17%), a 2-year college or undergraduate degree (65%), or graduate degrees (18%). They were employed in a variety of occupations, including hotel frontline employees (35%), marketing and sales (24%), education (14%), finance (8%), and others (19%). Detailed descriptions of the samples are presented in Table 1.

**Table 1:** Sample characteristics.

Measure	Items	Frequency (*n*)	Percentage (%)
Gender	Male	123	49
	Female	128	51
Education	High school diploma	42	17
	Associate degree	62	25
	Bachelor degree	100	40
	Master degree or above	47	18
Occupation	Hotel frontline employees	88	35
	Marketing and sales	60	24
	Education	35	14
	Finance	20	8
	Others	48	19

### Measures

The survey measurement was translated into Chinese from the original construct in English. Back-translation procedures (Brislin, 1980) were applied to improve measurement reliability and validity in a different language.

#### Job Complexity

We measured job complexity with the three-item scale developed by Shaw and Gupta (2004). One sample item is as: “*My job is very complex*.” Participants could respond to the items using a 5-point scale (1=*strongly disagree*; 5=*strongly agree*). The scale’s α=0.80.

#### Work Engagement

We used the nine-item version of the Utrecht work engagement scale (UWES; Schaufeli et al., 2006). The UWES items reflect three underlying dimensions, which are measured with three items each: Vigor (e.g., “*At my work, I feel bursting with energy*”); Dedication (e.g., “*I am enthusiastic about my job*”); and Absorption (e.g., “*I get carried away when I am working*”). All nine statements were measured with a 5-point Likert-type scale from 1(*strongly disagree*) to 5 (*strongly agree*). The scale’s α=0.93.

#### Energy Depletion

Adopting an item from Bakker and Oerlemans (2019), we measured energy depletion: “How much energy did your job cost you?” with a 5-point scale (1=*no energy whatsoever*; 5=*all of my energy*).

#### Job Crafting

We used the approach/avoidance crafting scale developed by Bruning and Campion (2018). Approach crafting was assessed using a 23-item scale (five dimensions, *α*=0.94), with items, such as “*Today, I expanded my role by providing opinions on important issues* (work role expansion),” “*Actively initiate positive interactions with others at work* (social expansion),” “*Create structure in my work processes* (work organization),” “*Use new knowledge or technology to enhance communication* (adoption),” and “*Use my thoughts to put myself into a good mood at work* (metacognition).” Avoidance crafting was measured using a two-dimension scale (*α*=0.90). An example item that reflects “work role reduction” is “*Find ways to get others to take my place in meetings*.” “Withdrawal” was measured with an example item, such as “*Work in a way that allows me to avoid others at work*.” Items were assessed with a five-point frequency scale where 1=*never* and 5=*all of the time*.

#### Control Variables

As previous research indicated that demographic variables, such as gender, age, education level, and tenure, could be related to job crafting (Tims et al., 2013; Rudolph et al., 2017), we controlled for these variables.

Important to check first is whether approach crafting and avoidance crafting are different constructs. The two-factor structure with approach and avoidance crafting as separate latent factors (*χ*^2^=58.075, *df*=53, *p*>0.05; CFI=0.997; RMSEA=0.020) provided a significantly better fit to the data than the single-factor structure (*χ*^2^=502.139, *df*=54, *p*<0.001; CFI=0.705; RMSEA=0.182; Δ*χ*^2^=444.064, *df*=1, *p*<0.001). Further, the correlation between the two job crafting measures observed in the current data (*r*=−0.08) was comparable with those reported in previous studies (Rudolph et al., 2017). Consistent with Rudolph et al. (2017), they meta-analytically examined how well the four job crafting dimensions proposed by Tims et al. (2012) fit together. The findings indicate that avoidance-oriented job crafting (i.e., decreasing hindering job demands) loaded much lower (0.047) than the other three approach-oriented job crafting dimensions (e.g., increasing social job resources).

### Analysis Strategy

To test this dual-mediation model, we used structural equation modeling (SEM) to investigate the hypothesized relationships using the Mplus software (v.8.3). Indirect effects were tested using the bootstrapping method. Following Edwards and Lambert (2007), we used moderated path analysis and bootstrapping to test the moderated mediation hypotheses. Given the relatively small sample size compared to the number of items, we modeled the variables as latent variables with parcels (Marsh et al., 1998). For example, for the latent construct approach crafting, five parcels were created for each approach crafting dimension. Parceling can make the measurement models more reliable (Little et al., 2002). Also, the reliability of the parcels was checked, and all reliability estimates were above 0.84.

## Result

### Reliability Analysis

Table 2 reports means, standard deviations, correlations, and their Cronbach’s alpha values of all study variables. The scales used were highly reliable as the value of Cronbach’s alpha was above the standard.70.

**Table 2:** Means, standard deviations, and correlations among study variables.

Variables	*M*	*SD*	1	2	3	4	5	6	7	8	9
1.Age	42.12	12.37	–								
2.Gender	0.51	0.50	−0.01	–							
3.Education	15.46	1.84	−0.08	0.10	–						
4.Tenure	6.95	6.15	0.45^**^	0.00	0.03	–					
5.Job complexity	3.94	0.74	−0.07	−0.01	0.06	−0.04	**(0.80)**				
6.Work engagement	3.86	0.80	−0.06	−0.03	0.06	0.05	0.18^**^	**(0.93)**			
7.Energy depletion	3.53	1.10	−0.01	−0.05	−0.17^**^	−0.06	0.40^**^	−0.11	–		
8.Approach crafting	3.61	0.72	−0.07	0.03	−0.11	−0.07	0.29^**^	0.25^**^	−0.05	**(0.94)**	
9.Avoidance crafting	3.59	0.93	0.01	0.02	−0.09	0.03	0.17^**^	−0.17^**^	0.43^**^	−0.08	**(0.90)**

*n=251. Gender (male=0; female=1); education (e.g., “12” for “high school diploma,” “15” for “associate degree,” “16” for “bachelor degree,” and “18” for “master degree or above”). Reliabilities of the scales are boldfaced and noted in the diagonals*.

** *p<0.01. Two-tailed tests*.

### Common Method Bia

Given that data were collected through a single source, we conducted Harman’s single-factor analysis to test whether common method bias can be a potential concern in our study (see Podsakoff and Organ, 1986). The first factor in the unrotated structure had an eigenvalue of 10.90 and only accounted for 25.96 per cent of the variance. This suggests that common method bias is less likely to influence the results of this study.

### Confirmatory Factor Analyses

Before hypotheses testing, we conducted a series of confirmatory procedures to examine the discriminant validity of the study’s key measures: job complexity, work engagement, approach, and avoidance crafting (see Table 3). We excluded energy deletion into our measurement model because the model failed to convergence (only one item scale to measure energy depletion). The results showed that proposed four-factor measurement model resulted in a good fit with the data (*χ*^2^=278.82, *df*=224, *p*<0.01; CFI=0.98; RMSEA=0.03). In addition, the standardized factor loadings ranged from 0.67 to 0.85, and all of them were significant at a level of *p*<0.001, demonstrating relatively good convergent validity. Table 3 shows that various alternative measurement models displayed a significantly worse fit than the hypothesized four-factor model (all Δ*χ^2^* tests, *p*<0.01). The results provided support for the distinctiveness of the key measures in this study. Thus, we proceeded to hypothesis testing using the five study variables.

**Table 3:** The confirmatory factor analysis results.

Model	Descriptions	*χ* ^2^	*df*	TLI	CFI	RMSEA	△*χ*^2^
Model l	Four factors: Job complexity, work engagement, approach crafting and avoidance crafting	278.82	224	0.98	0.98	0.03	
Model 2	Three factors: Job complexity and work engagement were combined into one factor.	547.96	227	0.89	0.90	0.08	269.15^***^
Model 3	Three factors: Approach crafting and avoidance crafting were combined into one factor.	755.36	227	0.81	0.83	0.10	476.54^***^

*** *p<0.001*.

### Hypotheses Testing

We tested our dual-research model in Mplus Version 8.3 (Muthén and Muthen, 2017). In Hypothesis 1, we expect that job complexity predicts approach crafting. The results showed that complex job was positively related to approach crafting (*b*=0.27, *p*<0.001; see Table 4), supporting Hypothesis 1. Hypothesis 2 suggests that job complexity is positively related to avoidance crafting. The results revealed that job complexity was positively related to avoidance crafting (*b*=0.22, *p*<0.001; see Table 4). Thus, Hypothesis 2 was supported.

**Table 4:** Path coefficients and indirect effects for mediation models.

	Point Estimate	Bootstrapping	
		Product of Coefficients		BC 95% CI	
		S.E.	Est./S.E.	Lower	Upper
**Effects from job complexity to approach crafting *via* work engagement**					
Total effects	0.27	0.06	4.46	0.15	0.39
Indirect effects	0.03	0.02	1.99	0.01	0.07
Direct effects	0.24	0.06	4.05	0.13	0.36
**Effects from job complexity to avoidance crafting *via* energy depletion**					
Total effects	0.22	0.09	2.53	0.07	0.40
Indirect effects	0.22	0.04	5.00	0.15	0.32
Direct effects	−0.00	0.09	−0.16	−0.25	0.18

Job complexity was significantly related to work engagement (*b*=0.18, *p*<0.05; see Figure 1), supporting Hypothesis 3. In Hypothesis 4, we expect that work engagement mediates the relationship between job complexity and approach crafting. We bootstrapped 10,000 samples and used the bootstrap estimates to construct bias-corrected confidence intervals (CI) for all significance tests reported in this study (Mooney et al., 1993; Shrout and Bolger, 2002). The bootstrap estimate (0.03) for the indirect effect fell within the 95% bias-corrected confidence interval 95% CI [0.01, 0.07], supporting the significance of work engagement as a mediator in the relationship between job complexity and approach crafting (see Table 4). As to Hypothesis 5, job complexity was significantly related to energy depletion (*b*=0.61, *p*<0.001; see Figure 1). In Hypothesis 6, the bootstrap estimate (0.22) for the indirect effect fell within the 95% bias-corrected confidence interval 95% CI [0.15, 0.32], supporting energy depletion fully mediating the relationship between job complexity and avoidance crafting (see Table 4). Thus, Hypothesis 6 was supported.

## Discussion

This study provided insight into how job complexity can lead to approach and avoidance crafting *via* two mediating pathways. The three-wave field study involving 251 employees revealed that job complexity was positively associated with work engagement and energy depletion, which in turn, were positively associated with approach and avoidance crafting, respectively. Our findings are consistent with previous findings which suggested that employees craft their jobs to acquire, protect, and retain resources to deal with stressful situations as they arise (Nielsen, 2013; Harju et al., 2016).

Our findings show that job complexity leads to avoidance crafting only when energy is depleted, while it is in any case directly related to approach crafting. The possible explanations are that individuals see potential gains in proactively changing job characteristics, such as job demands and resources (Ma et al., 2019). Individuals strive for problem-solving goals and take over the challenging tasks once they believe such challenges may fulfill their competence and autonomy (Parker et al., 2006). However, avoidance crafting may lower employees’ perception of self-competence and frustrate their positive self-image among others (Wang et al., 2017). Taking the interpersonal work context into account, coworkers reinforce the “approach” or reject the “avoidance” work behaviors (Tims and Parker, 2019). When job crafters withdraw from their work role under the complex jobs, coworkers’ may have negative attribution that may decrease the job crafter’s positive self-image. As such, our results show that job complexity directly leads to approach strategies, which may prevent energy depletion in the long term, while it leads to avoidance crafting only when energy is already depleted.

### Theoretical Implications

Our study makes several theoretical contributions to the field. Firstly, this study examines the underlying psychological mechanisms of job complexity and approach/avoidance crafting and contributes valuable knowledge to elucidate the psychological mechanisms linking the various antecedents to approach and avoidance crafting, a field of research that is only poorly explored (Ghitulescu, 2007). Individuals can either try to handle complex job demands by optimizing their resources investment through approach crafting, or they may try to cope with excessive demands by engaging in avoidance crafting (Zhang and Parker, 2019; Costantini et al., 2021). We suggest that the current resource state of an employee determines his/her adoption of approach versus avoidance crafting. Specifically, our study indicated that work engagement (i.e., resource gain process) and energy depletion (i.e., resource loss process) trigger approach and avoidance job crafting, respectively. This finding indicates that the emergence of different job crafting types from the same task design can instigate different resource states among employees.

Secondly, our results emphasize the importance of motivational and strain aspects. Complex jobs oriented on development stimulate higher levels of work motivation by creating critical psychological states in employees (i.e., work engagement) that enable them to engage in approach crafting. Job complexity leads to energy depletion which can lead to avoidance crafting. We therefore investigated how job complexity can trigger different employee workplace attitudes which can in turn prompt different forms of job crafting. Our results provide input for future research on the motivational and strain consequences of job complexity on employee functioning.

Thirdly, using COR theory as theoretical lens, our study offers empirical evidence that supports the resource gain and loss processes postulated in COR theory (Halbesleben et al., 2014). COR theory suggests that these competing processes help explain how individuals manage their resources in order to cope with demands (Dawson et al., 2016). However, prior studies mainly concentrated on the resource loss process, while neglecting resource gain (Hobfoll et al., 2018). The present study considers both processes in parallel, thereby facilitating a deeper understanding of both processes. Moreover, our results reveal that job complexity may display a “too-much-of-a-good-thing” effect, resulting in strain outcomes when taken too far (see Pierce and Aguinis, 2013, p. 315).

### Practical Implications

The present findings also hold practical implications for managers. Jobs are becoming increasingly complex as they often involve more flexible and fluid employment arrangements (Okhuysen et al., 2013). Managers should be aware that the constantly changing demands of today’s complex jobs cannot wait for top-down job design solutions (Grant and Parker, 2009; Demerouti, 2014). Rather, managers should recognize the importance of bottom-up work design approaches (i.e., job crafting) and initiate interventions to channel employee job crafting efforts to a desired direction. Our findings suggest that inducing work engagement among employees (e.g., vigor, dedication, and absorption) can effectively promote approach crafting. In contrast, employees’ energy depletion may lead to avoidance crafting. Thus, managers should carefully increase additional resources, such as job autonomy, to allow employees to master their complex tasks. In the context of a stressful and demanding work, employees craft their jobs to acquire and protect resources. Thus, organizations and managers should provide employees with opportunities to craft their jobs to achieve a better balance between job demands and resources, which will lead to positive outcomes (Kristof-Brown et al., 2005).

Furthermore, instead of focusing on the beneficial and motivational effects of job complexity, organizations also need to adopt a more balanced view that also considers the potential strain effects of challenging jobs. This means that managers should monitor employees and ensure that task demand is reasonable, and control the flow of demands to ensure sufficient activation and prevent the depletion of the employees’ physical and emotional resources. As such, both top-down job design and bottom-up job crafting have significant implications for individual and organizational performance (Demerouti et al., 2019; Lichtenthaler and Fischbach, 2019).

### Limitations and Directions for Future Research

The present study had several limitations that should be addressed in future research. Firstly, while we used self-reporting to assess the variables, which may lead to common method bias (Podsakoff et al., 2003), we adopt a potential procedural remedy in the form of three surveys conducted over a period of 4 weeks (Podsakoff et al., 2003). Our statistical analyses suggest that common method bias may not be a serious problem. Additionally, self-reported assessments of job crafting are appropriate because job crafting is self-initiated behavior. Thus, employees are in a better position than their supervisors and peers to rate their job crafting activities. Nevertheless, future research on job crafting may benefit from observing “actual job crafting,” e.g., measuring the amount of time spent on each task.

Secondly, although the dual pathway of the job crafting model is promising, other moderators, such as different personalities, should be explored to better understand when and how complex jobs influence job crafting. Future research could also examine the moderating effects of leader behavior on the job complexity-job crafting relationship (Thun and Bakker, 2018). Further, the potential moderating role of personal and job resources and their effects on job strain and work engagement should be explored in more detail (Bakker and Demerouti, 2017). Specifically, based on Job Demands and Resources theory, job resources (such as skill variety) and personal resources (such as self-efficacy) have a particular effect on motivation when job demands are high. Thus, employees with high job and personal resources may be more motivated to adopt job crafting.

Thirdly, our study does not consider the social, political, and relational elements of job characteristics which may influence how employees interpret their job tasks (Grant and Parker, 2009). Thus, future studies should examine whether other job-design characteristics are able to predict approach and avoidance crafting. Moreover, we did not consider the relationship between hindrance job demands (e.g., role conflict or role ambiguity) and job crafting. Therefore, future studies should examine a broader range of job demands to identify those that cause individuals to reduce and increase their job crafting efforts.

Fourthly, in an effort to avoid similarities and possible collinearities with work engagement, this study did not use the four items scale (Ryan and Frederick, 1997) to measure employees’ energy depletion. Instead, we adopted the item scale developed by Bakker and Oerlemans’s (2019) to assess employees’ positive energy available for purposive action. As this might cause validity issues, future research should consider other instruments to measure energy depletion, such as the 25-item state resource depletion scale by Ciarocco et al. (2007). Lastly, the extent to which our results can be generalized is unclear. Previous studies shown that low-skilled workers might implement job crafting differently from highly skilled professionals (Nielsen and Abildgaard, 2012). Future studies should verify our findings by using a more diverse group of individuals. Given that the average age of our sample was about 42 years, future research should examine and test our model in younger employees.

## Conclusion

We investigated the relationship between job complexity and job crafting through resource gain and resource loss processes based on COR theory. Whereas job complexity may lead to approach crafting through work engagement, it can also deplete employees’ energy, which may lead to avoidance crafting. The present study points out important research directions that can further expand our knowledge of the dual effects of job complexity on job crafting.

## Data Availability Statement

The raw data supporting the conclusions of this article will be made available by the authors, without undue reservation.

## Ethics Statement

The studies involving human participants were reviewed and approved by the Academic Committee, Macau University of Science and Technology. The patients/participants provided their written informed consent to participate in this study.

## Author Contributions

JB and QT contributed to this work equally. All authors contributed to the article and approved the submitted version.

## Conflict of Interest

The authors declare that the research was conducted in the absence of any commercial or financial relationships that could be construed as a potential conflict of interest.

## Publisher’s Note

All claims expressed in this article are solely those of the authors and do not necessarily represent those of their affiliated organizations, or those of the publisher, the editors and the reviewers. Any product that may be evaluated in this article, or claim that may be made by its manufacturer, is not guaranteed or endorsed by the publisher.
